# The Uvalde school shooter: uncovering the dreadful story behind an antisocial profile

**DOI:** 10.3389/fnbeh.2025.1593203

**Published:** 2025-08-29

**Authors:** Carlos Ramos-Galarza, Jennifer Obregón

**Affiliations:** Facultad de Salud y Bienestar, Carrera de Psicología Clínica, Pontificia Universidad Católica del Ecuador, Quito, Ecuador

**Keywords:** child abuse, animal cruelty, family violence, antisocial behavior, mass shooter

## Abstract

**Introduction:**

The case of the Texas shooter is an event that marked human history, as an 18-year-old young man cold-bloodedly murdered 21 victims.

**Objective:**

To analyze the psychological factors that could explain the antisocial behavior of the case under study.

**Methodology:**

Through clinical behavioral analysis and data collection from the case, a psychological profile was constructed to identify specific factors that provide greater clarity regarding the risk factors associated with the violent act, which enabled the development of this perspective article.

**Results:**

The study delves into several factors that could be considered determining and causal to the antisocial behavior in this case, including child abuse, family violence, bullying, animal cruelty, the impact of maltreatment on brain development, and the relationship between antisocial behavior and the use of violent video games.

**Discussion:**

The analysis concludes by emphasizing the need to identify the causal factors of antisocial behavior in the early stages of human development. Additionally, it highlights the importance of implementing early interventions that can positively influence the factors described in this article.

## 1 Introduction

The relationship between child abuse, family violence, animal cruelty, bullying, and neurodevelopmental issues can lead to a greater tendency toward antisocial behavior in individuals. This is exemplified by the case presented in this perspective article, a young man who experienced all the aforementioned factors and ultimately committed one of the most brutal acts of violence ever witnessed in an educational setting.

Below, we analyze the case of the Texas shooter, a young man who cold-bloodedly murdered children at an elementary school. In this analysis, we will address the trajectory of Ramos’ behavior that led to the mass shooting, with a focus on identifying key causal and contributory factors. Early influences include childhood abuse and exposure to family violence, which functioned as initial causal factors. Determining factors encompass a caregiver with substance use and their normalization of aggressive parenting, accompanied by a lack of support from other family members.

Maintaining factors involve frequent exposure to aggressive media through violent video game use and the perceived reinforcement from online communities that promote violent behaviors. Aggravating factors include persistent exposure to bullying and engagement in animal cruelty acts. These elements contributed to behavioral consequences such as neurodevelopmental issues, desensitization to violence, decreased empathy, and self-harming behavior, which will be described in more detail in the following paragraphs. While mass shootings are inherently complex and multi-determined, this categorization offers a structured framework for understanding the multifactorial origins of antisocial behavior.

In this perspective article, we adopted a methodology grounded in the clinical analysis of a single case. The primary theoretical framework guiding our work is neuropsychology, encompassing its clinical, developmental, and pathological subfields. It is essential to emphasize that the range of factors to which an individual is exposed during childhood can significantly influence brain development and organization, either positively or negatively. In the case analyzed, we hypothesize that exposure to multiple adverse experiences negatively impacted brain function, contributing to abnormal neuropsychological development, which ultimately manifested in severely disrupted behavior.

We hope this analysis will contribute to the dialog on preventing such behaviors in educational environments. By intervening early in any of the factors discussed below, it may be possible to prevent violent events like the one that occurred in Texas.

## 2 The perspective behind a mass murderer

### 2.1 The background of the shooter

The case analyzed in this study involves Salvador Ramos, an 18-year-old solitary individual who was a victim of bullying due to his linguistic difficulties from an early age and who experienced childhood abuse while growing up in a family environment marked by relational difficulties. His behavior was characterized by social isolation, involvement in altercations at school, and a disturbing tendency to mistreat animals, which he often shared on social media. On May 24, 2022, shortly after turning 18, Ramos purchased firearms and meticulously planned an attack on Robb Elementary School in Uvalde, Texas. During the commission of this heinous act, as evidenced by video recordings, Ramos acted with cold-blooded intent, murdering 21 individuals (19 students and 2 teachers) and injuring several others before being neutralized by local law enforcement. This tragic event underscores the devastating consequences of a life marked by trauma, isolation, and unchecked aggression.

Salvador Ramos, born on May 16, 2004, was an 18-year-old Hispanic individual who committed multiple homicides by firearm. His background includes a history of bullying, family violence, neurodevelopmental disorders, childhood maltreatment, animal abuse, self-harming behavior, aggression, family disorganization, threats of sexual violence, school dropout, and a mother with drug use (Swissinfo, 2022; RFI, 2022; El País, 2022).

### 2.2 Child abuse and family violence

Although there is limited information about Ramos’ early childhood, it is stated that during his adolescence, he had an absent father and abusive and neglectful treatment from his mother (Stevens, 2022). Based on a report to the FBI from a former girlfriend, she believed Ramos had been sexually abused by one of his mother’s boyfriends during his childhood. However, when he confided in his mother about the incident, she did not believe him (Public Broadcasting Service News, 2022).

Also, some testimonies mentioned that Ramos’s mother used to do drugs and was rarely available and that he once uploaded a video to a social media story in which he was shouting at and verbally insulting his mother as she expelled him from the household. Ramos experienced these kinds of disturbances numerous times, one of which involved police officers arriving on the day he relocated to his grandmother’s residence (Economic Times, 2022; Ortiz, 2022).

### 2.3 Struggles with bullying

Ramos faced difficult experiences with school harassment starting from his fourth-grade year. According to reports from his family members and classmates, he was frequently ridiculed for his strong lisp and stutter, which affected his speech. He was also bullied for aspects such as his physical appearance, his often-repetitive daily attire, and his hair length, even being called a “school shooter” (Public Broadcasting Service News, 2022; Economic Times, 2022). On one occasion, after posting a photo of himself wearing black eyeliner, he was subjected to ridicule through a derogatory term targeting homosexual individuals. These experiences led to him struggling to get along with his classmates, having few friends, missing extended periods of high school, and engaging in self-harm behavior, such as cutting up his face (O'Rourke, 2022).

Other reports suggest that Ramos was a bully himself. For instance, in the years preceding the deadly attack, he discharged an air gun at unfamiliar individuals and hurled eggs at vehicles. Additionally, multiple individuals reported his Yubo account due to its explicit content and lascivious threats, one of which included a threat of sexual violence against a female user. Also, one of his classmates informed that he instigated conflicts with others and deliberately attempted to disturb individuals at the park (O'Rourke, 2022; Sarkar, 2022).

### 2.4 Animal cruelty

According to several users of the streaming platform Yubo, Ramos engaged in acts of animal abuse in public, primarily directed toward cats, often laughing with friends and boasting about doing it frequently. As stated by them, he repeatedly live-streamed these acts on this social media, one of which included footage of himself holding a plastic bag containing a dead cat. Based on the testimony of one of his classmates, Ramos was once observed beating a dog (Sarkar, 2022).

Animal abuse has been linked to multiple forms of aggressive and antisocial behavior. In children and adolescents, it has been associated with conduct disorders and an increased likelihood of committing recurrent acts of interpersonal violence in adulthood (Hensley et al., 2018). Regarding family violence, in households where any form of violence is present, the probability of animal abuse occurring is substantially heightened (McPhedran, 2009; Degue and Dilillo, 2009). Experiencing bullying has also been related to animal torture; specifically, both experiencing and perpetrating physical bullying demonstrated the highest rates of involvement in multiple acts of animal abuse, as well as the lowest levels of sensitivity toward attitudes related to animal cruelty (Henry and Sanders, 2007).

Animal cruelty, in this case, may not be a causal factor per se but rather a behavioral manifestation of underlying antisocial tendencies. It serves as an early warning sign of conduct pathology and may contribute to the desensitization process, thereby increasing the likelihood of future violent behavior.

Furthermore, repeated exposure to violence against animals can alter brain function in individuals who engage in or witness such acts. This desensitization to violence is associated with changes in brain structures, such as the amygdala, which may increase in size and exhibit altered functioning. These changes can impair the ability to process and respond appropriately to others’ emotions. As a result, some individuals who commit violent acts may display a lack of empathy or compassion toward their victims, thereby increasing the risk of repeated aggressive behavior (van Rooij et al., 2020; Cacciaglia et al., 2017).

Another brain structure negatively affected by constant exposure to violence, such as animal cruelty, is the prefrontal cortex. This region plays a crucial role in executive functions, particularly in inhibitory control, which is essential for regulating automatic or impulsive behaviors (Ramos-Galarza and Pérez-Salas, 2017). Repeated exposure to violent stimuli may impair the development and functioning of the prefrontal cortex, leading to a diminished capacity to suppress harmful behaviors (Hummer et al., 2019).

In the case of Ramos, at the moment he likely felt the impulse to kill, his prefrontal cortex was not functioning adequately. This dysfunction may have hindered his ability to inhibit such a destructive action, contributing to the tragic outcome.

### 2.5 The impact of child maltreatment on brain development

To understand the antisocial behavior of an individual such as the Texas gunman, it is essential to consider that childhood maltreatment represents an etiological factor associated with brain damage. This is because current research indicates that individuals exposed to high levels of stress during early developmental stages often exhibit elevated cortisol levels, which can lead to irreversible impairments in brain function (Şimşek et al., 2016).

In the abuse experienced by the analyzed case, situations are described in which the individual endured physical, psychological, and sexual violence, creating a detrimental context for normal brain development. Such harm is evident in individuals through impairments in behavioral skills, including emotional and behavioral self-regulation, cognitive difficulties in memory, attention, and executive functions, as well as a diminished capacity to make appropriate decisions (Cellini, 2004).

In the case under analysis, the maltreatment experienced during childhood led to brain alterations that created a negative predisposition for the development of antisocial behavior, such as endangering the lives of other human beings. For instance, it has been reported that childhood maltreatment can result in a smaller brain size due to the reduction of gray matter caused by the effects of abuse (Yang et al., 2023). This alteration generates lower intellectual functioning, learning difficulties, reduced cognitive performance, and impairments in emotional regulation (Joseph et al., 2023; Hart and Rubia, 2012).

### 2.6 Is there a relationship between antisocial behavior and the use of violent video games?

The relationship between antisocial behavior and the use of violent video games is a complex and controversial topic that remains under debate. However, in the analyzed case, it is noteworthy that the individual, through his engagement with various video games centered around weapon-based killings, threatened to carry out real-life massacres after losing several matches (Prensa Libre, 2022).

Previous research has indicated that engagement with violent video games is associated with an increase in aggressive and antisocial behavior. Specifically, the use of violent video games that simulate the use of firearms has been identified as a potential predictor of anger and physical aggression among high school students, as exemplified by the case under analysis (Farrar et al., 2017; De Medeiros et al., 2020). The use of video games has also been linked to desensitization to violence, reduced empathy, and a lower likelihood of exhibiting prosocial behavior. This form of desensitization may contribute to acts of antisocial behavior; however, current research does not establish a direct correlation with criminal acts (Brockmyer, 2015; Fraser et al., 2012). As evidenced, there is a clear relationship between the use of violent video games and aggressive behavior, as demonstrated in this case. For example, previous research has found that playing violent video games is associated with decreased functioning of the prefrontal cortex (Hummer et al., 2019). Nonetheless, further research is still necessary to explore its connection with antisocial behavior. It is important to emphasize that, in the case under examination, other determining and causal factors of antisocial behavior are present, resembling a chain of events in which the final link is the massacre.

## 3 Discussion

The case analyzed in this perspective article involves an 18-year-old individual whose early life was marked by severe maltreatment, including physical, psychological, and sexual abuse, as well as neglect. Such adverse experiences cause lasting alterations in brain development, particularly in regions responsible for emotional regulation, memory, and decision-making. Chronic stress and elevated cortisol levels likely impaired the prefrontal cortex, hippocampus, and amygdala, contributing to difficulties in emotional self-regulation and cognitive functioning. These neurological impairments, combined with persistent bullying and social isolation, shaped a volatile psychological profile. Ridiculed for his speech impediment, appearance, and clothing, the individual experienced profound social withdrawal and feelings of inadequacy. Notably, being labeled a “school shooter” by peers may have reinforced a distorted self-perception, further exacerbating his antisocial tendencies. Despite being a victim of bullying, he also exhibited aggressive behaviors himself, including threats and acts of animal cruelty, illustrating the cyclical nature of violence, where trauma often begets aggression.

The individual’s documented acts of animal cruelty, shared on social media, served as a significant warning sign, aligning with research that links animal abuse to violent behavior toward humans. These acts likely desensitized him to violence, normalizing it as an outlet for his anger. This behavior, coupled with his history of abuse, bullying, and family dysfunction, created a dangerous trajectory. His mother’s drug use and failure to protect him from abuse fostered a chaotic and unsafe home environment. At the same time, the absence of a stable parental figure left him without guidance or emotional support. The apparent dismissal of his allegations of sexual abuse further deepened his isolation and mistrust, compounding his trauma. Although his engagement with violent video games may have reinforced aggressive tendencies, it is crucial to note that such media alone are unlikely to cause antisocial behavior. Instead, they may have acted as one of many contributing factors within a broader context of risk.

This case underscores the cumulative effect of multiple risk factors—childhood maltreatment, bullying, family dysfunction, animal cruelty, and exposure to violent media—in shaping violent behavior. While no single factor thoroughly explains his actions, their interplay created a perfect storm culminating in tragedy. This highlights the critical need for early intervention and comprehensive support systems to address these interconnected risks. Accessible mental health services, targeted interventions for at-risk youth, and efforts to break cycles of violence are essential in preventing similar tragedies. By understanding the complex interplay of factors that contribute to violent behavior, we can better identify and support vulnerable individuals before their behavior escalates.

## Data Availability

The original contributions presented in the study are included in the article/supplementary material, further inquiries can be directed to the corresponding author/s.
